# Microlearning and online simulation-based virtual consultation training module for the undergraduate medical curriculum – a preliminary evaluation

**DOI:** 10.1186/s12909-023-04777-1

**Published:** 2023-10-25

**Authors:** Siaw Cheok Liew, Maw Pin Tan, Emer Breen, Kuhan Krishnan, Inthirani Sivarajah, Nivashinie Raviendran, Thidar Aung, Amal Nimir, Vinod Pallath

**Affiliations:** 1 Medical Education Research and Development Unit, Faculty of Medicine, Universiti Malaya, Kuala Lumpur, Malaysia; 2 Department of Clinical Competence, Perdana University-Royal College of Surgeons in Ireland, Kuala Lumpur, Malaysia; 3 VinUniversity, Hanoi, Vietnam; 4 Department of Medicine, Faculty of Medicine, Universiti Malaya, Kuala Lumpur, Malaysia; 5 Dean’s Office, Faculty of Medicine, Universiti Malaya, Kuala Lumpur, Malaysia; 6 Department of Pathology, Manipal University College Malaysia, Malacca, Malaysia

**Keywords:** Virtual consultation, Telemedicine, Medical students, Medical training, Course evaluation

## Abstract

**Background:**

Virtual consultation is a synchronous mode of telemedicine provided remotely via information and communication technology. The projected growth of digitalization in healthcare delivery, however, necessitates medical student training in virtual consultation (VC) to ensure safe and effective patient care. This study describes the implementation and preliminary evaluation of a competency-based VC training module for undergraduate medical students.

**Methods:**

A newly developed six-week VC module was implemented online through asynchronous microlearning and synchronous simulation-based experiential learning modalities. Clinical students in years 4 and 5 and fresh graduates, who had not started pre-registration house officer or residency programmes, were invited to participate. Training outcomes using checklist-based video-recorded assessments of VC encounters between medical students and simulated patients were compared. Each video was independently assessed by two facilitators trained in VC teaching and assessment, using a direct observed virtual consultation skills checklist derived from established VC competencies. The participants completed course evaluations electronically as additional outcome measures.

**Results:**

Fifty-two clinical phase medical students and alumni completed both the instructional and practical phases of this module. Altogether, 45 (95.7%) students found the module beneficial, and 46 (95.9%) reported increased self-efficacy for conducting VC. In total, 46 (95.9%) students would recommend the course to others. Post-test results showed a significant increase in the students’ abilities to conduct a VC (t-test = 16.33, *p* < 0.05).

**Conclusion:**

Microlearning and simulation-based sessions were effective instructional delivery modalities for undergraduate medical students in their attainment of VC competencies.

**Supplementary Information:**

The online version contains supplementary material available at 10.1186/s12909-023-04777-1.

## Background

The use of telemedicine has grown exponentially in recent years, from the use of telephones for primary healthcare-related consultations to e-visits for specialised follow-up, for instance, in mental health-related issues [1]. There are four types of telemedicine: (i) synchronous video and telephone consultations that provide real-time patient care, (ii) asynchronous messaging systems used between physicians and patients, for example, the use of email, (iii) remote monitoring of patient health data, for example, remote blood pressure monitoring, and (iv) the use of mobile health apps to promote positive health behaviour communication between the health provider and patients [2]. Virtual consultation (VC) is a synchronous mode of telemedicine and is defined as a medical service provided remotely via information and communication technology [3].

The coronavirus disease pandemic has propagated telemedicine use, and it is likely to remain a healthcare delivery model, with proven benefits for equity in healthcare and patient satisfaction [4]. This novel and rapid expansion of telemedicine has training implications for clinical staff and medical students. In 2021, the Association of American Medical Colleges (AAMC) and the Accreditation Council for Graduate Medical Education (ACGME) developed telemedicine competencies for graduates, residents, and physicians that should be applicable to medical students and which could be used as part of their continual assessment [2]. With the pressing need to include VC training, medical educators face challenges introducing this into existing undergraduate curricula. Therefore, innovative approaches are needed to develop opportunities to do this within the rigid structures of existing curricula. Despite initiatives to include VC training in undergraduate teaching since the 1990s, there remains a lack of educational research informing best practices for medical student training in VC.

This module was introduced entirely online, supplemented by microlearning in its instructional phase and followed by online simulation-based learning in its experiential learning phase. The application of microlearning synergises with the concept of a just-in-time learning model, whereby focused, tailor-made lessons can be delivered immediately [5]. This opportunity is usually offered outside of traditional classroom learning and serves as a supplementary tool for more formal exposure to experiential learning, as in a practical session [6]. Previous studies have reported the effectiveness of microlearning in healthcare professions [7, 8]. A scoping review reported that participation in microlearning sessions provided improvement in students’ knowledge and confidence when performing procedural skills [7]. Despite its popularity in health disciplines, microlearning has not knowingly been described as a feature in the curriculum design for the instructional phase of any VC module.

There is a lack of published evidence on the effective delivery of VC training despite its increasing use in health care. This study describes the development of a VC training module and discusses the insights gained from an evaluation of its implementation, developed using Kern’s six-step model [9]. The research findings should help address the current non-availability of a VC training module and elucidate its perceived effectiveness. By ensuring comprehensive implementation and evaluation, we can help promote patient safety and secure the future of VC in healthcare.

## Methods

### Study participants

This module was implemented as a pilot study that aimed to train senior medical students (year 4 and 5 medical students and fresh medical graduates who had not yet started their housemanship or residency programme) who were recruited through consecutive sampling. All available students and alumni that met the inclusion criteria were invited to participate. Invitations were sent through email, word of mouth, and social media. The students were expected to have acquired basic skills in patient-doctor communication, history taking, and physical examination before being eligible to participate in the module. Participation in the study was voluntary and written informed consent was obtained from all participants. To ascertain with 95% certainty that an issue would be detected in the pilot study if the problem manifested itself with at least 0.05 probability (i.e., in at least 1 out of 20 participants), a total of 60 participants was considered an adequate sample size [10].

### Module development

**Fig. 1:**
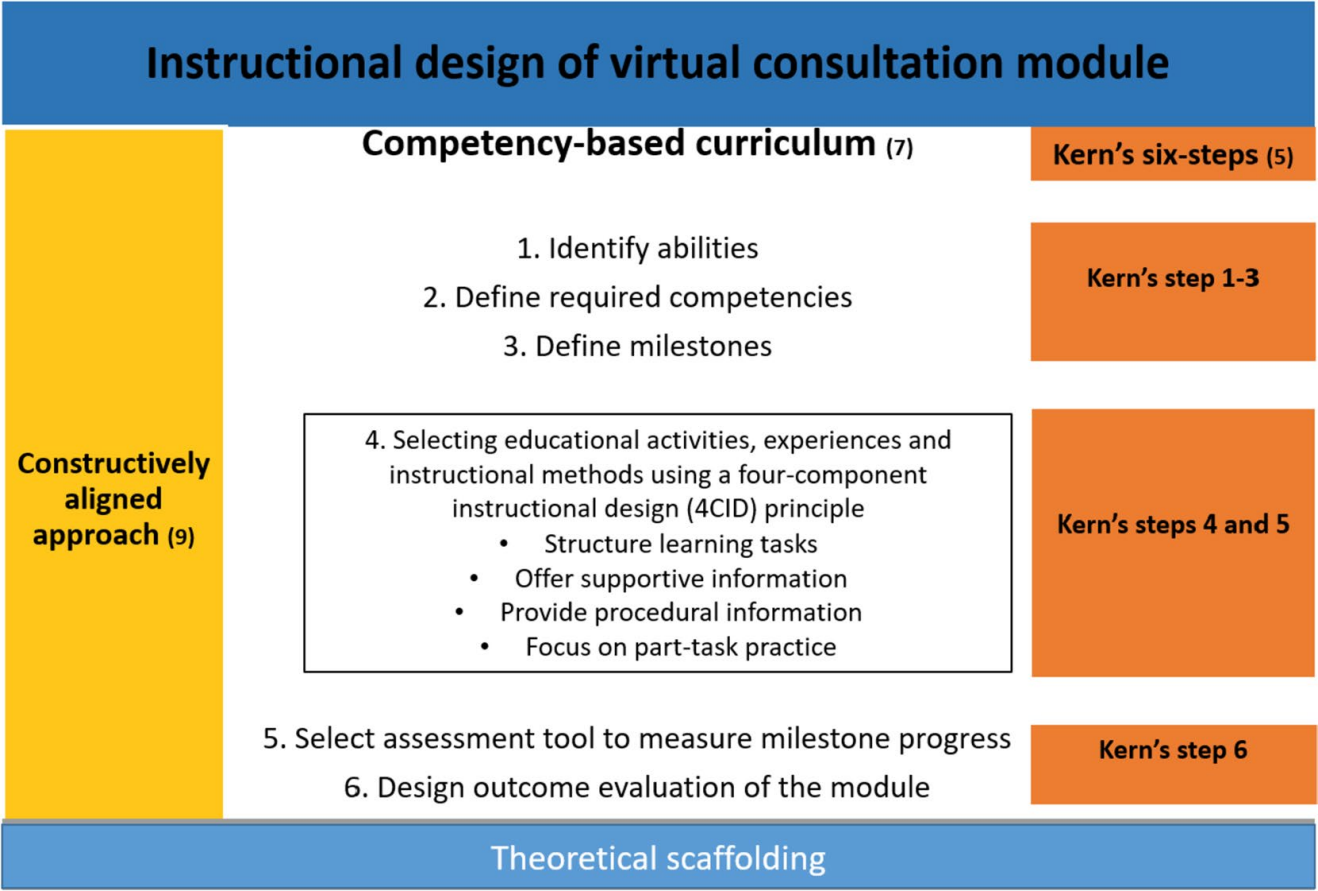
Theoretical scaffolding of the instructional design for the module. The figure illustrates the instructional design of the VC module, conceptualised through Kern’s six-step model, which leverages on the principles of a competency-based curriculum. The module was constructively aligned with the teaching and learning activities and an assessment framework

This paper describes steps 4, 5, and 6 of Kern’s six-step model (see Fig. 1) in VC module development, using a competency-based approach. Competency-based medical education (CBME) helps health professionals develop skills for specific contexts through learner-centred approaches [11]. The introduction of entrustable professional activities (EPAs) allows the translation of competencies into practice. In addition, EPAs support the execution of tasks and responsibilities, once the trainee has achieved the desired competencies [12]. The research team determined an educational strategy, designed an educational module (Kern’s steps 4 and 5), implemented it in a pilot study, and evaluated its effectiveness (Kern’s step 6) in undergraduate medical students [9].

The module design was scaffolded using CBME. Constructive alignment [13] was applied to consolidate the teaching–learning and assessment strategies in the four-component instructional design (4CID) principles of simulation-based education (SBE) [14]. The 4CID model in SBE encompasses the following: (i) structuring learning tasks, (ii) offering supportive information, (iii) providing procedural information, and (iv) focusing on part-task practice [14]. This approach helps ensure patient safety in addition to that provided by clinical scenario simulation. Evidence supports that SBE best practice includes the introduction of a range of difficulties with clinical variation; the allowance for deliberate, distributed, and repetitive practice; and the opportunity for feedback in individualised learning, leading to mastery [15]. In the constructive alignment approach [13], these teaching and learning activities and assessments were aligned with the VC competencies. Additional file 1 shows the constructive alignment of the VC module. Table 1 shows the summary of module activities delivered over six weeks.

**Table 1:** Summary of learning and assessment in the virtual consultation module

	**Syllabus topic**	**Learning opportunities/ instructional delivery method**	**Assessment format and purpose**	**Personnel involved**
**WEEK 1 (pre-test)**	• Learners attempt a VC with an SP using prior knowledge of how to conduct a medical consultation	• Pre-test	• DOVCS assessment • SP feedback	• SP
**Instructional Phase**				
**WEEK 1**	• Initiating a VC • History-taking and effective communication • Ethics and professionalism	• Microlearning: videos 1–5 • Completion of SP script for role play (due end of the week)	• Quiz (Formative) • Peer feedback (Formative)	• Self- directed learning • Peer for role play
**WEEK 2**	• Observational examination • Managing a patient • Concluding a VC	• Microlearning: videos 6–9 • Completion of SP script for role play (due end of the week) • Review two given papers and submit a reflective report	• Quiz (Formative) • Peer feedback (Formative)	• Self- directed learning • Peer for role play
**Experiential Learning Phase**				
**WEEK 3**	• Initiating a VC • History-taking and effective communication • Ethics and professionalism	• Facilitated with simulated patient practice sessions • One-on-one SP encounter	• Peer feedback (Formative) • SP feedback (Formative) • Facilitator feedback (Formative)	• Facilitator • SP • Peers
**WEEK 4**	• Observational examination	• Facilitated with SP- practice sessions • One-on-one SP encounter	• Peer feedback (Formative) • SP feedback (Formative) • Facilitator feedback (Formative)	• Facilitator • SP • Peers
**WEEK 5**	• Managing a patient • Concluding a VC	• Facilitated with SP- practice sessions One-on-one SP encounter	• Peer feedback (Formative) • SP feedback (Formative) Facilitator feedback (Formative)	• Facilitator • SP • Peers
**WEEK 6 (post-test)**	Learners attempt a VC with a SP	• One-on-one SP encounter	• DOVCS assessment • SP feedback (Summative)	• SP

*Abbreviation*: *VC* virtual consultation, *SP* simulated patient, *DOVCS* Directly observed virtual consultation skills

### Module delivery

**Fig. 2:**
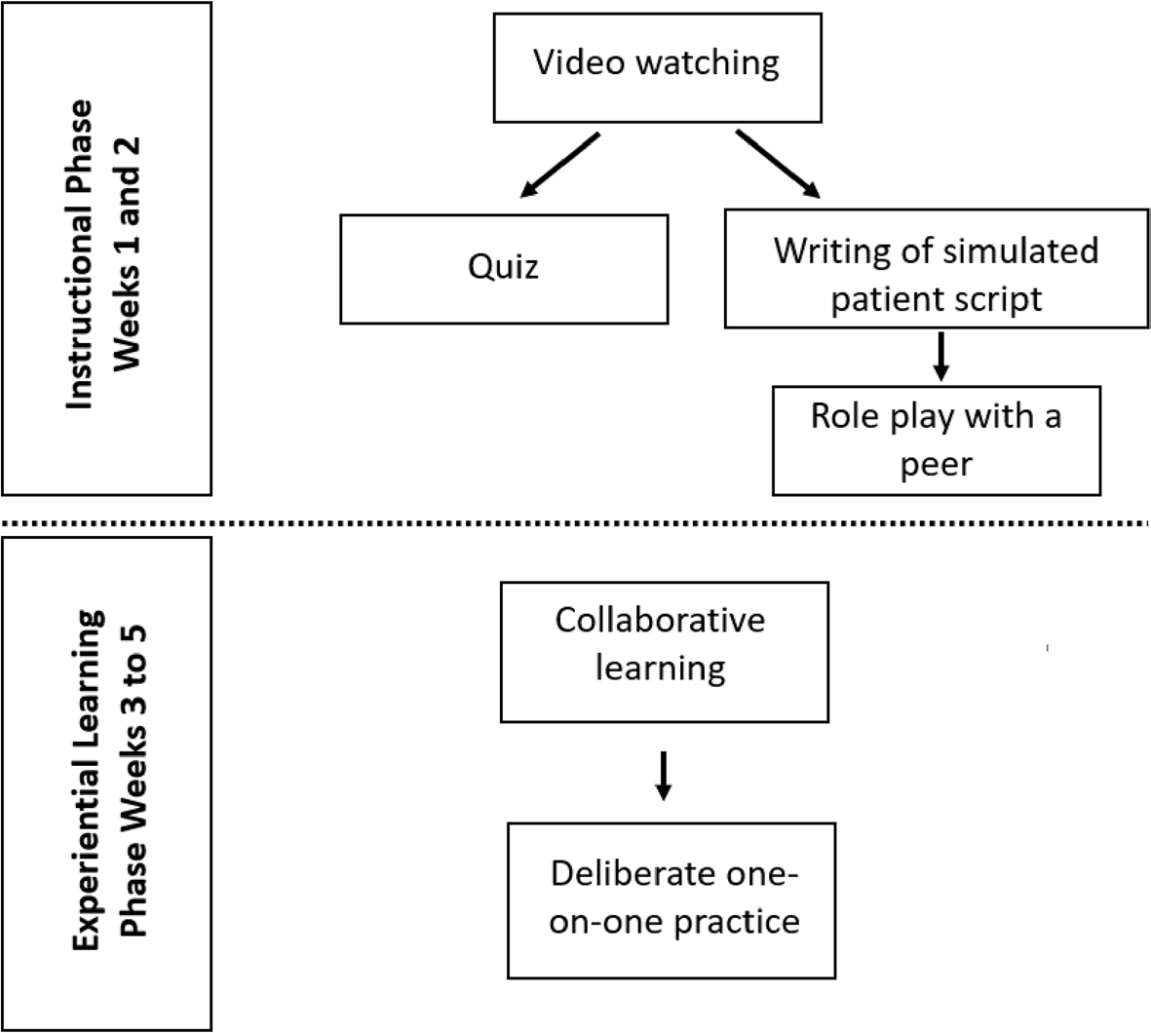
Flowchart of the module delivery

The module was delivered in two phases: (i) instructional and (ii) experiential learning. Weeks 1 and 2 (instructional) were intended for knowledge acquisition and weeks 3 to 5 (experiential) consisted of practical VC sessions. Week 6 consisted of a post-test assessment. Figure 2 shows the phases of module delivery.

For the instructional phase, supportive information was provided through microlearning. This is an innovative pedagogy that embraces emerging technology to deliver small bite-sized lessons and activities, which help consolidate the achievement of specified learning objectives [16]. Asynchronous lessons, using this technique, ensured rapid learning, allowing lessons to be accessed at the learner’s convenience. During the experiential learning phase, medical students were given the opportunity to learn in structured practical sessions, in which weekly practical sessions were segmented by their focus on different VC skill sets. In keeping with experiential learning theory, the practical phase (or the synchronous sessions) of this VC module was designed to give the medical students, in groups of five or six and supported by a VC-trained facilitator, the opportunity to conduct VCs on simulated patients (SPs). Over three weeks, the medical students, a facilitator, and an SP met once a week online, for 1.5 h a session, to practice history taking, observational examination, and management of a patient through a VC. Immediate feedback from peers, facilitators, and SPs encouraged improvement in competency acquisition. Following this, each medical student individually conducted a 25-min VC session with the SP, allowing deliberate practice of skills learnt.

### Assessments for and of learning

(i) Formative assessments and opportunities for feedback

After watching videos on each of the topics (see Table 1) delineated during the instructional phase, participants were required to answer quizzes on a learning management system, Moodle^TM^ (Moodle HQ, Perth, Australia). The quizzes were between five to ten questions, using Google Forms (Mountain View, California, United States). Peer-led role plays were conducted at the end of weeks 1 and 2 to help students to consolidate learning that they had received from the instructional phase. In the experiential learning phase, facilitated peer-assisted collaborative learning gave opportunities for feedback from the facilitator, peer, and SP. Furthermore, the deliberate practice session allowed participants to receive additional feedback from the SPs. Figure 2 shows the flowchart for the instructional approaches to the module.

(ii) Summative assessment: Pre- and post-testing using a workplace-based assessment tool

In the pre- and post-test sessions, the medical students were invited to participate in a 20-min direct observed virtual consultation skills (DOVCS) assessment session before the start of the asynchronous session in week 1 and at the end of the synchronous sessions in week 6. In these DOVCS sessions, medical students were expected to take a history, perform an observational examination, and manage the SP before concluding the VC. The sessions were conducted and recorded via a commercially available video conferencing platform (Zoom™, San Jose, United States). Each recorded video was given to two faculty members to watch and evaluate independently, using a DOVCS checklist (Additional file 2) developed by the research team, to assess the medical students’ VC competencies. The mean scores gathered from each reviewing pair were collated for analysis. Faculty members, who were also the facilitators in the experiential learning (synchronous) sessions and examiners for the DOVCS sessions, were required to watch all instructional training videos designed for the medical students prior to attending a face-to-face, online training session. In this session, they were provided with training on conducting a VC, including a discussion on the competencies required. Prior to each teaching week 3, 4, and 5, a further online training sessions was conducted with the faculty members by one of the core research team members, well-versed in the competencies and methods of module delivery. Any concerns arising from previous sessions were discussed, and instructions for the delivery of the next medical student session were provided to ensure standardisation of teaching and assessment.

### Direct observed virtual consultation skills checklist

The DOVCS checklist was developed by the research team based on the VC-EPA competency framework (Additional file 2). The checklist comprised a four-point assessment instrument: 0 = not performed, 1 = needs improvement, 2 = satisfactorily performed, and 3 = excellent performance. Altogether, there were 13 items in the DOVCS checklist, and the possible total score was 39 marks. The themes, as determined by the VC-EPA competency framework, comprised elements of: pre-consultation preparation, interview initiation, history-taking performance, observational examination performance, patient management, and VC conclusion.

### Evaluation of the virtual consultation module

New World Kirkpatrick’s model Level 1 (Reaction) and Level 2 (Learning) were applied in the evaluation of the module’s effectiveness [17, 18]. The medical student’s module evaluation was gathered through feedback collected mid-way (after the asynchronous microlearning sessions), repeated at the end of the practical (synchronous) sessions, and after attending the post-test DOVCS assessment. Feedback was sought from SPs and facilitators at the end of the module. A pre-test and post-test study design was employed to assess whether learning outcomes for the VC module were achieved.

The medical students evaluated the module anonymously using a Google Form (Mountain View, California, United States). In the evaluation of the asynchronous phase, a 5-point Likert rating scale was employed in response to statements regarding the medical student ‘s experience of using Moodle™ (Moodle HQ, Perth, Australia), the quality of videos and quizzes, and the time allocated for the learning sessions. In addition, medical students rated the overall delivery of each phase of the module on a scale between 0 (not favourable) to 10 (excellent). Similar scales were used to evaluate the synchronous phase, assessing the student’s learning experiences with the SP and faculty member. In the overall module evaluation, medical students assessed module content, adequacy of time given for learning, and quality of training received. In addition, the students expressed their opinions of the module, by describing what sessions they found valuable, areas of concern, and suggestions for improvement.

Faculty members and SPs were also given the opportunity to provide anonymous module feedback through an evaluation survey at the end of the module, using a 5-point Likert rating scale (1 = strongly disagree, 2 = disagree, 3 = neither agree nor disagree, 4 = agree, 5 = strongly agree), which was also completed electronically.

### Data analysis and statistics

Prior to data entry, all completed DOVCS checklists and module feedback from the medical students, SPs, and faculty members were cross-checked for completeness. Statistical analysis was conducted using the Statistical Package for the Social Sciences (SPSS) Version 20.0 (IBM™, Armonk, USA). Continuous data are presented using mean and standard deviation with categorical data displayed using frequency and percentage. The mean pre-test and post-test DOVCS evaluation scores were compared using paired t-tests. A p-value of < 0.05 was considered statistically significant.

### Ethics approval

The study was approved by the Medical Research Ethics Committee of Universiti Malaya Medical Centre (MRECID.NO: 2021130–9777) and Perdana University Institutional Review Board (PUIRB 325). The research was conducted in accordance with the Declaration of Helsinki.

## Results

### Demographic characteristics of participants

The students were recruited from 1^st^ August to 30th September 2022, following which the study was conducted from September to December 2022. A total of 136 students and alumni were invited to participate, from which sixty-two consented to join. Ten participants (16.1% attrition rate) dropped out prior to the implementation phase due to personal or family issues, and some students found it difficult to juggle their studies with course completion. A total of 16 (30.8%) Year 4 students, 17 (32.7%) Year 5 students and 19 (36.5%) graduates participated in this study.

### Kirkpatrick Level 1 (Reaction) post instructional microlearning

The feedback sought at the end of week 2, following completion of the microlearning instructional asynchronous phase, revealed that 48 (100%) of the participants (*n* = 48/52, response rate 92.3%) intended to apply the knowledge and skills gained during the experiential learning sessions. Positive feedback scores were received for all feedback items. A total of 44 (91.7%) agreed that they felt more confident in their ability to deliver a VC, and 46 (95.9%) stated that they would recommend this learning module to others. The overall rating scores (out of 10) of the instructional phase were 6 and above. The mean score was 8.44 (SD 1.15) with the breakdown of the scores attained as follows: score 6 (4.2%), 7 (16.7%), 8 (33.3%), 9 (22.9%), and 10 (22.9%).

### Kirkpatrick Level 1 (Reaction) post experiential learning (practical) phase

The feedback sought from participants at the end of week 5, after the completion of the practical synchronous phase, revealed that all respondents, 48 (100%) (response rate 48/52, 92.3%), were confident in their ability to perform VC sessions with patients. Positive feedback scores were received on the feedback items listed. Altogether, 46 (95.9%) of the participants stated that they would recommend the course to others. The overall rating scores (out of 10) of the practical phase were 5 and above. The mean score was 8.63 (SD 1.36) with the breakdown of the scores attained as follows, 5 (2.1%), 6 (6.3%), 7 (12.5%), 8 (20.8%), 9 (22.9%), and 10 (35.4%).

### Kirkpatrick Level 1 (Reaction) overall module feedback

The overall module feedback was obtained from 47 medical students, a response rate of 90.4%. Positive feedback scores were received for all items in the feedback form. Medical students were generally satisfied with the facilitator, SP, course content, course delivery, and allocated time for learning. Table 2 shows the aggregated feedback scores. The rating scores (out of 10) of the overall module evaluation were 5 and above. The mean score was 8.53 (SD 1.21) with the breakdown of the scores attained as follows, 5 (2.1%), 6 (2.1%), 7 (14.9%), 8 (27.7%), 9 (27.7%), and 10 (25.5%).

**Table 2:** Kirkpatrick Level 1 (Reaction) core evaluation feedback for overall module delivery

Question	Strongly disagree n (%)	Disagree n (%)	Neither agree nor disagree n (%)	Agree n (%)	Strongly agree n (%)
**Medical Students (** ***N*** ** = 47)**					
Q1. The expected outcomes of the VC course were clear to me	0 (0.0)	0 (0.0)	0 (0.0)	25 (53.2)	22 (46.8)
Q2. The module was well organised	0 (0.0)	1 (2.1)	0 (0.0)	26 (55.3)	20 (42.6)
Q3. The videos were well prepared and easy to follow	1 (2.1)	1 (2.1)	0 (0.0)	18 (38.3)	27 (57.4)
Q4. Moodle ™ was well organised and easy to follow	0 (0.0)	0 (0.0)	2 (4.3)	26 (55.3)	19 (40.4)
Q5. The content of the learning material was relevant to a VC	0 (0.0)	1 (2.1)	0 (0.0)	20 (42.6)	26 (55.3)
Q6. The time given for learning was sufficient to achieve the competencies required to deliver a VC	0 (0.0)	1 (2.1)	4 (8.5)	19 (40.4)	23 (48.9)
Q7. I was given sufficient materials to support learning to achieve the competencies required to deliver a VC	0 (0.0)	1 (2.1)	2 (4.3)	20 (42.6)	24 (51.1)
Q8. I was given sufficient opportunities to learn and practice to achieve the competencies required to deliver a VC	0 (0.0)	0 (0.0)	0 (0.0)	20 (42.6)	27 (57.4)
Q9. Overall, I found the module beneficial	0 (0.0)	2 (4.3)	0 (0.0)	16 (34.0)	29 (61.7)
Q10. I am satisfied with the delivery of the module	0 (0.0)	0 (0.0)	1 (2.1)	19 (40.4)	27 (57.4)
Q11. The facilitator in my group has helped me to achieve the competencies required for VC delivery	0 (0.0)	0 (0.0)	0 (0.0)	14 (29.8)	33 (70.2)
Q12. The simulated patients (overall) have helped me to achieve the competencies required for VC delivery	0 (0.0)	0 (0.0)	2 (4.3)	12 (25.5)	33 (70.2)
**Simulated patients (** ***N*** ** = 9)**					
Q1. The expected outcomes of the module were clear to me	0 (0.0)	0 (0.0)	0 (0.0)	2 (22.2)	7 ( 77.8)
Q2. The module was well organised	0 (0.0)	0 (0.0)	0 (0.0)	3 (33.3)	6 ( 66.7)
Q3. I had access to sufficient materials to support my role as an SP in this course	0 (0.0)	0 (0.0)	0 (0.0)	2 (22.2)	7 ( 77.8)
Q4. The learners were given sufficient time to practice the VC skills	0 (0.0)	0 (0.0)	0 (0.0)	3 (33.3)	6 ( 66.7)
Q5. The conduct of the practical sessions in weeks 3–5 were relevant to the VC course	0 (0.0)	0 (0.0)	0 (0.0)	0 ( 0.0)	9 (100.0)
Q6. The content of the practical sessions in weeks 3–5 were relevant to the VC course	0 (0.0)	0 (0.0)	0 (0.0)	1 (11.1)	8 ( 88.9)
Q7. The learners were more competent to deliver the VC module towards the end of the training	0 (0.0)	0 (0.0)	0 (0.0)	1 (11.1)	8 ( 88.9)
Q8. The VC course is effective in enabling the development of VC competencies to learners who are medical students, house officers and medical officers	0 (0.0)	0 (0.0)	0 (0.0)	0 ( 0.0)	9 (100.0)
Q9. I am satisfied with the delivery of the VC course	0 (0.0)	0 (0.0)	0 (0.0)	3 (33.3)	6 (66.7)
**Faculty (** ***N*** ** = 4)**					
Q1. The expected outcomes of the VC module were clear to me	0.0	0.0	25.0	50.0	25.0
Q2. The module was well organised	0.0	0.0	0.0	50.0	50.0
Q3. The content of the instructional phase (videos, quiz) on Moodle ™ was relevant to the VC course	0.0	0.0	0.0	50.0	50.0
Q4. The content of the instructional phase (videos, quiz) on Moodle ™ was easy to follow	0.0	0.0	0.0	75.0	25.0
Q5. The conduct of the practical sessions in weeks 3–5 was relevant to the VC course	0.0	0.0	0.0	50.0	50.0
Q6. The content of the practical sessions in weeks 3–5 was relevant to the VC course	0.0	0.0	0.0	25.0	75.0
Q7. The VC course had sufficient materials to support learners' achievement of competencies to deliver a VC	0.0	25.0	0.0	50.0	25.0
Q8. The VC course had sufficient allocation of time to support learners' achievement of competencies to deliver a VC	0.0	0.0	0.0	25.0	75.0
Q9. The VC course had sufficient opportunities for learners to learn and practice to support learners' achievement of competencies to deliver a VC	0.0	0.0	0.0	25.0	75.0
Q10. The VC course is effective in enabling the development of VC competencies to learners who are medical students, house officers, and medical officers	0.0	0.0	0.0	50.0	50.0
Q11. I am satisfied with the delivery of the VC course	0.0	0.0	0.0	100.0	0.0

*Abbreviation*: *VC* virtual consultation

### Feedback from simulated patients and facilitators

Table 2 shows the SP responses about the module. Ten SPs were recruited for the study. One SP dropped out from the module and nine SPs continued to contribute to the VC module delivery. All nine SPs (100%) responded to the feedback request. The overall rating scores (out of 10) were 7 and above. All nine (100%) SPs thought that the expected outcomes of the module were clearly specified, the module was well organised, they had access to sufficient materials to support their role, the learners were given sufficient time to practice the VC skills, the practical sessions were relevant, the learners were more competent at the end of their training, the course was effective in enabling VC competency, and they were satisfied with the overall course delivery.

A total of four faculty members were trained to deliver the experiential learning (practical) sessions as a facilitator, all four contributed to the delivery of the VC module, and four (100%) provided feedback. The overall rating scores (out of 10) for the VC module were 8 and above. The mean score was 8.50 (SD 0.58); two faculty members gave a score of 8 (50.0%) and two a score of 9 (50.0%). All four faculty members (100%) thought that the module was well organised and effective for enabling the development of VC competencies in medical students and were satisfied with the delivery of the VC module. Table 2 shows the facilitator responses.

### Kirkpatrick Level 2 (Learning) pre-test and post-test evaluation

(i) Validity and reliability of the DOVCS checklist

The DOVCS checklist had an internal consistency of 0.92 with inter-rater reliability determined by an intraclass coefficient of 0.68, and 95% confidence intervals between 0.201 and 0.901, p < 0.001, based on a mean-rating (k = 2), absolute-agreement, 2-way mixed-effects model.

(ii) Pre- and post-test analysis

A total of 52 students participated in the pre-test and post-test DOVCS. The pre-test and post-test DOVCS evaluation showed a significant improvement in the medical students’ scores in the post-test assessment compared with the pre-test (t-score = 16.33, *p* < 0.05). The mean pre-test score was 13.22 (SD = 3.4) and mean post-test score was 23.42 (SD = 4.4). A total of 45 (86.5%) medical students scored higher than the minimum 50% passing mark in the post-test assessment. The highest score that a participant achieved in the post-test was 75.6% and the lowest was 26.9%. The mean difference in the median scores for the pre-test and post-test assessments was 10.20 (SD 4.51).

## Discussion

This paper describes the development and implementation of a VC training module. The design of a training module for the purpose of competency development needs to be grounded in, and supported by, relevant conceptual models. In this study, the CBME concept and the 4CID model provided this guidance. The constructive alignment principle was used to identify and develop comprehensive learning opportunities. The content of the module was based on the outcome competencies developed by the research team, following the CBME approach. This rigorous evidence-based process of competency development resulted in the construction of explicit task lists and comprehensive narration of outcome competencies. This validated and clearly articulated framework of competencies enabled the identification of syllabus content, learning opportunities, and instructional approaches used in the module; thus, validity of the content and process of development was ensured. The New World Kirkpatrick Model was used to assess the effectiveness of the module [18]. Results show that the participants found microlearning in the instructional and stimulation-based experiential learning phases satisfactory and beneficial (Reaction; Kirkpatrick’s Level 1), and almost all of them would recommend the module to others. The post-test scores indicated an improvement in the medical students’ VC knowledge and skills (Learning; Kirkpatrick’s Level 2). These pilot study findings indicate that participants accepted the module and achieved the desired competencies. The real test of the module will be whether it can enable the students to carry out independent, unsupervised VC clinical practice. Such an analysis is beyond the scope of this study.

The implementation phase of a CBME module also needs evaluation, as the process of implementation is as important as the outcome, in addition to the evaluation of participant outcomes [19]. Quality of implementation significantly influences CBME outcomes. Constructive alignment of numerous educational elements including intended learning outcomes, learning opportunities and assessment strategies must operate in sync to facilitate the participant's attainment of competence. Van Melle et al. (2019), on behalf of the International Competency-based Medical Education Collaborators, developed a core component framework for evaluating the effectiveness of CBME programme implementation [19]. This includes five core components: (i) clearly articulated outcome competencies, (ii) competencies sequenced progressively indicating logical development, (iii) tailored learning experiences aligning to the progressive achievement of competencies, (iv) competency-focused instruction enabling the sequential achievement of competencies, and (v) programmatic assessments supporting the achievement of competencies.

An evaluation of the VC module, through the lens of a core component framework, showed that it had clearly articulated outcome competencies, making the task clear and specific. The competencies progressed sequentially from initiating the VC, history taking, observational examination, management, and conclusion. Learning experiences were sequential and supported the achievement of mastery learning. The flexible learning and online-based design of the module encouraged student participation. As the module was conducted in tandem with usual clinical training, medical students were glad that the module provided flexibility for them to learn online and in their own time. The scheduling of sessions during weekends and evenings facilitated learner participation. There were opportunities for feedback and refinement of practice during the assessments. Evaluation results obtained from this course mirror previous evidence, which proposes microlearning as a valuable tool to supplement learning, especially before macrolearning, such as a didactic lecture or practical session [20]. The flexibility and ease of learning through this platform made it easier for medical students to grasp the concept of VC in their own time and space [21]. Self-determined learning (heutagogy) was crucial for the medical students who had to balance their workload. Microlearning provided up-to-date, in-the-moment information to students, helping ensure knowledge and foundational grounding in VC practice, hence building confidence before practical sessions.

The opportunity for experiential learning through simulation, during the practical VC sessions with facilitators and SPs, significantly boosted medical students’ self-efficacy in VC. This study highlights the importance of collaborative learning in skill acquisition, whereby participants learn together with peers through observation and active participation in practical sessions [22]. This study also highlights the importance of deliberate practice as students were given extra one-on-one practice with the SPs, followed by immediate SP feedback. The simulated learning in VC skill acquisition supports Vygotsky’s theory around “the zone of proximal development”, whereby guidance, demonstration, and feedback were given by trained faculty members and SPs, who were more experienced than the medical students in VC practice [23]. The context in which Vygotsky’s theory is discussed here highlights the potential development of those inexperienced in VC to achieve desired competencies, by being guided by those who are more knowledgeable. Vygotsky also advocated for collaborative learning environments, which help to enhance cognitive development through shared experiences and perspectives [23].

The students recruited were from the senior years of medical school, who had prior exposure to clinical skills training for face-to-face consultations. The optimal timing of VC training is yet to be determined. Introducing this in the second half of the medical curriculum, during the clinical rather than pre-clinical phase, implies that VC skills are adapted from basic clinical skills. Introducing VC earlier in the undergraduate curriculum, during the pre-clinical phase, could reinforce the pivotal role that VC is likely to play in future clinical practice and highlights that it requires specific rather than adapted skills. The research team postulates that the same microlearning content would be applicable to junior students (years 1 and 2), because the content of the instructional phase was based on the processes of VC practice; hence, applicable to any level of learning. Some medical schools may express reservations with introducing VC training early, as there is a prior need for a fundamental understanding of history taking, ethics, and physical examination. For junior students, simulation cases can be tailored to their learning needs. The medical cases discussed in the practical session could be purposively chosen to include different specialities and cater for increasing complexity as learners progress.

Facilitators who graded the DOVCS post-test assessments may have also taught the students in the synchronous phase of the learning, which could have biased the results. This was minimised by having two independent facilitators evaluate each student and the average scores obtained. While a pre- and post-test design can provide valuable insights to the effectiveness of a module, shortcomings of this approach must be considered. The lack of a control group means it is difficult to determine if the improvement observed in the post-test scores were solely due to the intervention received or whether other factors. e.g., remembering pre-test questions or response-shift bias, were at play. Immediate improvements experienced by the participants, may not predict long-term effects. Another important limitation of this study was the lack of real patient encounters. This limitation can be offset by incorporating clinical-based training in VC.

The introduction of VC skills through micro-credentialing courses as part of continuous learning for postgraduate students should be explored. Additional research is required to examine the long-term impact of VC training on students' skills and confidence, the different methods of VC training and their effectiveness, the training methods required for differing medical specialties, and the development and validation of tools to assess medical students' VC skills.

## Conclusions

An effective VC module was developed and implemented within a well-established medical programme. Further work is needed to determine whether this model could be fine tuned for use in junior students and across the curriculum. Ongoing evaluation is required to continuously improve this VC module and be assured of its sustainability as the most acceptable training format. These findings should be validated through replication of this research in other student groups across diverse institutions.

### Supplementary Information

**Additional file 1.** Constructive alignment of the virtual consultation module.

**Additional file 2.** Direct observed virtual consultation skills (DOVCS) checklist.

## Data Availability

The datasets used and/or analyzed during the current study are available from the corresponding author upon reasonable request.
