# Supplementary material for: Microlearning and online simulation-based virtual consultation training module for the undergraduate medical curriculum – a preliminary evaluation
Source: BMC Med Educ. 2023 Oct 25;23:796. doi: 10.1186/s12909-023-04777-1 (PMC10601318; doi:10.1186/s12909-023-04777-1)
Supplement: Supplementary file 1 — Additional file 1. Constructive alignment of the virtual consultation module. [file 12909_2023_4777_MOESM1_ESM.docx]

**Additional file 1: Constructive alignment of the virtual consultation module**

|  | **Syllabus topic** | **Learning opportunities/ instructional delivery method** | **Assessment format and purpose** | **Competency alignment** | **Personnel involved in teaching and learning** |
| --- | --- | --- | --- | --- | --- |
| **WEEK 1 (Day 1)** | Learners attempt a VC with a SP using prior knowledge of medical consultations. | - Pre-test | - DOVCS assessment (Formative) | None | - Learner (Self) |
| **WEEK 1 (Day 2)** | Initiating a VC   - Pre-consultation preparation (video 1) - Initiating a VC (video 2) | - Microlearning: videos 1&2 - Completion of SP script for role play (due end of the week) | - Quiz (Formative) | - Demonstrate adequate pre-consultation preparation - Demonstrate technical proficiency in basic virtual communication and computing skills - Effectively initiate a VC | - Learner (Self) |
| **WEEK 1 (Day 3)** | History-taking and effective communication   - Effective communication in a VC (video 3) - Listening skills in a VC (video 4) - History taking in a VC (video 5) | - Microlearning: videos 3,4,&5 - Completion of SP script for role play (due end of the week) | - Quiz (Formative) | - Demonstrate effective communication skills - Demonstrate effective listening skills - Demonstrate effective use of collateral information - Perform comprehensive history taking to develop differential diagnoses - Display a patient-centred approach | - Learner (Self) |
| **WEEK 1 (Day 4)** | Ethics and professionalism   - Professionalism and ethics in a VC (video 6) | - Watch video 6 - Completion of SP script for role play (due end of the week) | - Quiz (Formative) | - Demonstrate consistent ethical and professional practice | - Learner (Self) |
| **WEEK 1 (Day 5)** | - Initiating a VC - History-taking and effective communication - Ethics and professionalism | - Role play with peer | - Peer feedback (formative) | - All competencies delivered in week 1 | - Peer |
| **WEEK 2**  **(Day 1&2)** | - OE in a VC (video 7) | - Watch video 7 - Read and review two given papers and submit a reflective report - Completion of SP script on OE for role play (due end of the week) | - Quiz (Formative) | - Perform OE (inspection) (within the scope and limit of observational examination in the clinical context) | - Learner (Self) |
| **WEEK 2**  **(Day 3)** | Managing a patient   - Managing a patient in VC (video 8) | - Watch video 8 - Read and review two given papers and submit a reflective report. - Completion of SP script on OE for role play (due end of the week) | - Quiz (Formative) | - Effectively convey the diagnosis to patients - Effectively communicate management plan to patients - Suggest a concise follow-up plan for patients - Explain thoroughly to patients, the collection of health-related data at home for monitoring and diagnosis - Perform an appropriate VC specialist referral as and when needed | - Learner (Self) |
| **WEEK 2**  **(Day 4)** | Concluding a VC   - Concluding a VC (video 9) | - Watch video 9 - Review two given papers and submit a reflective report. - Completion of SP script on OE for role play (due end of the week) | - Quiz (Formative) | - Effectively conclude a VC - Maintain a complete electronic medical record for every VC | - Learner (Self) |
| **WEEK 2**  **(Day 5)** | - OE - Managing a patient - Concluding a VC | - Role play with peer | - Peer feedback (Formative) | - All competencies delivered in week 2 | - Peer |
| **WEEK 3** | - Initiating a VC - History-taking and effective communication - Ethics and professionalism | - SP practice sessions - One-on-one SP encounter | - Facilitator   feedback   - Peer feedback - SP feedback (Formative) | - All competencies delivered in week 1 | - Peers - Facilitator - SPs |
| **WEEK 4** | - OE | - SP practice sessions - One-on-one SP encounter | - Facilitator feedback - Peer feedback - SP feedback   (Formative) | - Perform OE (inspection) (within the scope and limits of OE in the clinical context) | - Peers - Facilitator - SPs |
| **WEEK 5** | - Managing a patient - Concluding a VC | - SP practice sessions - One-on-one SP encounter | - Facilitator feedback - Peer feedback - SP feedback   (Formative) | - Effectively convey the diagnosis to patients - Effectively communicate management plan | - Peers - Facilitator - SPs |
| **Pre-test and Post-test** | - Initiating a VC - History-taking and effective communication - Ethics and professionalism - OE - Managing a patient - Concluding a VC | - One-on-one SP encounter | - DOVCS assessment - SP feedback (Summative) | - ALL competencies in week 1 to 4 | - SP |

Abbreviation: VC, virtual consultation; SP, simulated patient; DOVCS, Directly Observed Virtual Consultation Skills; OE, observational examination
