# Supplementary material for: Microlearning and online simulation-based virtual consultation training module for the undergraduate medical curriculum – a preliminary evaluation
Source: BMC Med Educ. 2023 Oct 25;23:796. doi: 10.1186/s12909-023-04777-1 (PMC10601318; doi:10.1186/s12909-023-04777-1)
Supplement: Supplementary file 2 — Additional file 2. Direct observed virtual consultation skills (DOVCS) checklist. [file 12909_2023_4777_MOESM2_ESM.docx]

**Additional file 2: Direct observed virtual consultation skills (DOVCS) checklist**

|  | **Not performed** | **Needs Improvement and unsatisfactory** | **Satisfactorily**  **performed** | **Excellent performance** |
| --- | --- | --- | --- | --- |
| **Pre-consultation analysis**   - Analyses pre-consultation patient data in the virtual system to prepare for the virtual consultation session | _missed a majority of the components of the task_ | _tasks performed but missed critical components_ | _tasks performed with all the critical components elicited satisfactorily but requires practice for mastery_ | _mastery in performance and shows ability to adapt to situation/context_ |
| **Initiating a virtual consultation (VC) (consent)**   - Ensures that the patient’s identity is confirmed - Ensures that the physician’s identity (Full Name), role, place of practice, and registration status are made known to the patient - Obtains consent from patients - Ensures that the identities of all other participant(s) (if any) involved in the VC encounters are disclosed to the patient and approved by the patient - Demonstrates preparedness to escalate emergency care during VC, if needed | _missed a majority of the components of the task_ | _tasks performed but missed critical components_ | _tasks performed with all the critical components elicited satisfactorily but requires practice for mastery_ | _mastery in performance and shows ability to adapt to situation/context_ |
| **Initiating a VC (setting the agenda for VC)**   - Reviews the scope and limitations of VC use - Sets expectations of a VC encounter | _missed a majority of the components of the task_ | _tasks performed but missed critical components_ | _tasks performed with all the critical components elicited satisfactorily but requires practice for mastery_ | _mastery in performance and shows ability to adapt to situation/context_ |
| **History Taking**   - Asks focussed questions to gather relevant information - Formulates a list of accurate differential diagnoses - Ensures accuracy of history gathered by summarising the information taken | _missed a majority of the components of the task_ | _tasks performed but missed critical components_ | _tasks performed with all the critical components elicited satisfactorily but requires practice for mastery_ | _mastery in performance and shows ability to adapt to situation/context_ |
| **Use of collateral information**   - Asks for the relevant collateral information from a caregiver (if any) - Analyses the existing information presented (file, discharge summary, home monitoring results) by patients/caregivers correctly | _missed a majority of the components of the task_ | _tasks performed but missed critical components_ | _tasks performed with all the critical components elicited satisfactorily but requires practice for mastery_ | _mastery in performance and shows ability to adapt to situation/context_ |
| **Effective communication skills**   - Displays effective verbal and non-verbal communication - Observes the patient’s verbal and non-verbal cues and responds appropriately - Pauses, listens and allows the patient to speak - Addresses the patient’s ideas, concerns and expectations (ICE) with empathetic responses | _missed a majority of the components of the task_ | _tasks performed but missed critical components_ | _tasks performed with all the critical components elicited satisfactorily but requires practice for mastery_ | _mastery in performance and shows ability to adapt to situation/context_ |
| **Observational examination**   - Assesses a patient’s ability and safety to perform the tasks - If needed and with consent, utilises caregiver to support a patient - Instructs the patient to perform correct movements or tasks - Gathers the relevant information through observational and appropriate communication | _missed a majority of the components of the task_ | _tasks performed but missed critical components_ | _tasks performed with all the critical components elicited satisfactorily but requires practice for mastery_ | _mastery in performance and shows ability to adapt to situation/context_ |
| **Communicating management plan**   - Communicates the need for the patient to be seen at a physical facility if the VC encounter is deemed inadequate - Evaluates the need to prescribe or provide treatment recommendations to the patient - Involves the patient in the decision-making for treatment - Initiates treatment for the patient, done in the best interest of the patient | _missed a majority of the components of the task_ | _tasks performed but missed critical components_ | _tasks performed with all the critical components elicited satisfactorily but requires practice for mastery_ | _mastery in performance and shows ability to adapt to situation/context_ |
| **Suggest a concise follow-up plan**   - Communicates a follow-up plan to every patient - Ensures adherence to the same obligations for patient follow-up as would be expected for a physical consultation - Analyses the need for a VC specialist referral | _missed a majority of the components of the task_ | _tasks performed but missed critical components_ | _tasks performed with all the critical components elicited satisfactorily but requires practice for mastery_ | _mastery in performance and shows ability to adapt to situation/context_ |
| **Explain thoroughly to patients, the home monitoring data collection**   - Communicates to the patient/caregiver the need for home monitoring and record-keeping of data collected - Educates the patient/caregiver on the home monitoring devices and the record-keeping processes | _missed a majority of the components of the task_ | _tasks performed but missed critical components_ | _tasks performed with all the critical components elicited satisfactorily but requires practice for mastery_ | _mastery in performance and shows ability to adapt to situation/context_ |
| **Effectively conclude a VC**   - Summarises the findings from a VC encounter to the patient - Addresses the patient’s queries. - Establishes a follow-up plan with every patient to ensure safety netting - Concludes a VC encounter by allowing the patient to disconnect first to ensure all queries are addressed | _missed a majority of the components of the task_ | _tasks performed but missed critical components_ | _tasks performed with all the critical components elicited satisfactorily but requires practice for mastery_ | _mastery in performance and shows ability to adapt to situation/context_ |
| **(If applicable) Demonstrate technical proficiency**   - Troubleshoots if technology fails to work - Demonstrates confidence when troubleshooting - Knows whom to contact to assist if troubleshooting fails - Has contingency plans if troubleshooting fails | _missed a majority of the components of the task_ | _tasks performed but missed critical components_ | _tasks performed with all the critical components elicited satisfactorily but requires practice for mastery_ | _mastery in performance and shows ability to adapt to situation/context_ |
| **Demonstrate consistent ethical and professional practice throughout a VC**   - Explains digital confidentiality and security to the patient - Maintains privacy and confidentiality of the patient by ensuring that conversation is not overheard or overseen, similar to the requirement seen in a physical consultation. - Wears appropriate attire - Demonstrates respect for the patient (for example, use of appropriate salutation) - Demonstrates adherence to patient autonomy in decision-making and choice of medical care - Demonstrates adherence to other ethical principles like beneficence, justice and non-maleficence, if applicable | _missed a majority of the components of the task_ | _tasks performed but missed critical components_ | _tasks performed with all the critical components elicited satisfactorily but requires practice for mastery_ | _mastery in performance and shows ability to adapt to situation/context_ |
